# Increased Risk of Osteoporotic Vertebral Compression Fractures Following Epidural Steroid Injections in Patients with Lumbar Degenerative Disease: A Retrospective Cohort Study

**DOI:** 10.3390/jcm13216379

**Published:** 2024-10-24

**Authors:** Hao-Wen Chen, Wen-Tien Wu, Chia-Ming Chang, Tzai-Chiu Yu, Ing-Ho Chen, Kuang-Ting Yeh

**Affiliations:** 1 Department of Orthopedics, Hualien Tzu Chi Hospital, Buddhist Tzu Chi Medical Foundation, Hualien 970473, Taiwan; charliechen728@tzuchi.com.tw (H.-W.C.); timwu@tzuchi.com.tw (W.-T.W.); fresheric@tzuchi.com.tw (C.-M.C.); feyu@tzuchi.com.tw (T.-C.Y.); ihchen@tzuchi.com.tw (I.-H.C.); 2 Institute of Medical Sciences, Tzu Chi University, Hualien 970374, Taiwan; 3 School of Medicine, Tzu Chi University, Hualien 970374, Taiwan; 4 Graduate Institute of Clinical Pharmacy, Tzu Chi University, Hualien 970374, Taiwan

**Keywords:** spinal stenosis, epidural injection, osteoporotic fractures, lumbar vertebrae, glucocorticoids

## Abstract

**Background/Objectives**: Lumbar degenerative disease is a common age-related condition, with epidural steroid injection (ESI) being a widely employed conservative treatment approach. However, the potential effect of ESI on osteoporosis and fracture risk remains unclear. This study investigated the risk of osteoporotic vertebral compression fractures (OVCFs) in patients with lumbar degenerative disease who underwent ESI treatment. **Methods**: A cohort of 64 patients who received ESI treatment and a control group of 256 patients were included in this study. Demographic data, clinical characteristics, and follow-up information were collected. Cox proportional hazards models were used to analyze risk factors for OVCF, and subgroup analyses were conducted. **Results**: OVCF was more common in the ESI group than in the control group (hazard ratio [HR]: 3.49, 95% confidence interval [CI]: 1.06–11.43, *p* = 0.039). After confounding factors were adjusted for, ESI remained an independent risk factor for OVCF (HR: 4.60, 95% CI: 1.01–20.89, *p* = 0.048). In a subgroup analysis, lower socioeconomic status was associated with higher OVCF risk (HR: 11.82, 95% CI: 1.06–131.26, *p* = 0.044). The ESI group had improved short-term pain relief, with nonsignificant long-term effects. **Conclusions**: Patients with lumbar degenerative disease receiving ESI treatment are at an increased risk of OVCF, particularly those with lower socioeconomic status. These findings underscore the importance of regular bone density monitoring and fracture prevention following ESI treatment. Clinicians should carefully weigh the short-term benefits of ESI against the long-term risks and develop individualized follow-up plans for high-risk patients.

## 1. Introduction

Lumbar spondylosis (LS), a degenerative condition of the lumbar spine, is increasingly common as populations age [1,2,3,4,5]. In addition to conventional conservative treatments such as oral medications and physical therapy, epidural steroid injection (ESI), which involves the introduction of a corticosteroid-contained formulation into the epidural space of the lumbar spine, has emerged as a popular nonsurgical option for managing intractable neurological symptoms from moderate to severe LS [6,7,8,9,10]. ESI involves the administration of corticosteroids into the epidural space of the lumbar spine, providing temporary pain relief. Despite its widespread use, concerns remain about the long-term effects of corticosteroid injections on bone health.

Osteopenia and osteoporosis, characterized by decreased bone mineral density (BMD) and impaired bone microstructure, are frequently associated with systemic corticosteroid use and can lead to osteoporotic fractures, including osteoporotic vertebral compression fractures (OVCFs), proximal femur fracture, proximal humeral fracture, and distal radius fractures [11,12,13,14,15]. Osteopenia and osteoporosis often share several predisposing factors with LS and are notoriously underdiagnosed and undertreated until fractures occur, resulting in considerable quality of life (QOL) and economic burdens [16,17,18,19,20,21,22,23,24,25]. Given the overlap in risk factors for LS and osteoporosis, the potential impact of ESIs on fracture risk has become a critical area of investigation.

While ESIs are effective in managing pain and neurological symptoms, the potential negative impact of corticosteroids on bone health remains unclear. Some studies have suggested that ESIs do not significantly increase the risk of fractures. For example, a large cohort study involving 7197 patients found no association between cumulative corticosteroid injection doses and an increased fracture risk, even in those with preexisting osteoporosis, indicating that concerns about fracture risk should not preclude the use of ESIs [26]. However, other studies have indicated an association between corticosteroid injections, such as ESIs, and an increased risk of osteoporosis and subsequent fractures. For example, a nationwide retrospective cohort study revealed that patients receiving ESIs for LS exhibited a significantly higher risk of osteoporosis than those who did not receive ESIs, with an absolute standardized hazard ratio of 1.23 [27]. Similarly, another study observed that each successive lumbar epidural steroid injection (LESI) increased the risk of vertebral fractures by 1.21 times, suggesting that repeated injections could exacerbate bone fragility [28].

Conversely, some evidence, including Medicare data analysis, has indicated an association between ESIs and a reduced risk of osteoporotic spine fractures, although this finding may not be clinically significant [29]. The contradictory results from different studies underscore the complexity of this relationship. Case studies and reviews have highlighted the importance of careful risk assessment when prescribing corticosteroids, particularly for long-term or repeated use, due to the potential for severe complications such as OVCFs [30,31,32,33]. The effect of corticosteroid injections on osteoporotic changes in the thoracolumbar spine remains an open question.

This study aimed to investigate the risk of OVCFs in patients receiving ESIs as a conservative nonsurgical treatment for LS. By conducting a retrospective cohort study at a medical center, we sought to clarify the relationship between ESI and osteoporotic fracture risk, providing valuable insights for clinicians managing patients with lumbar degenerative disease.

## 2. Materials and Methods

The study protocol was approved by the Institutional Review Board and Ethics Committee of Hualien Tzu Chi Hospital. A retrospective cohort study was conducted by enrolling patients who visited the outpatient departments (OPDs) of orthopedics, neurosurgery, and pain at Hualien Tzu Chi Hospital between 2020 and 2022 with symptoms of lumbar degeneration. Patients included in the study were men aged ≥50 years and postmenopausal women. Exclusion criteria included congenital spinal pathology, previous spine surgery, or a history of osteoporosis-related trauma. Patients were selected based on subjective complaints of low back pain, radicular pain, or any neurological disorder. Neural and physical examinations were conducted by qualified specialists in orthopedics, neurosurgery, and pain. The diagnosis of LS was confirmed through radiological findings on plain films, computed tomography (CT), or magnetic resonance imaging (MRI). Data for qualified patients were extracted and documented in a database.

### 2.1. Epidural Steroid Injections

Patients who received ESIs as a nonsurgical treatment for LS were identified from the database to form the ESI group. The indications of ESI were patients that could not tolerate either oral medication, physical therapy, or lifestyle changes for the existing symptoms of lumbar degeneration for at least three months and meanwhile had not gone through any spinal surgery for the degenerative pathology yet. The date of the ESI was considered the index date. The ESIs were performed by qualified physicians via a caudal tunnel of the spine, through fluoroscopy-guided or echo-guided approaches, or both. The injected formula consisted of local anesthesia (1% lidocaine or 2% lidocaine) and corticosteroids such as dexamethasone, betamethasone, or triamcinolone, depending on the physician’s preference.

### 2.2. Propensity Score Matching

To reduce selection bias and approximate a randomized trial, a 1:4 propensity score matching (PSM) was performed. This propensity score is a single value between 0 and 1 that represents the relationship between multiple characteristics and the dependent variable. In this case, the PS predicted the probability of receiving an ESI. The four control samples matched on PS were similar to each ESI case in terms of all the covariates and demographics used to calculate the PS. The final matched-pair samples comprised closely matched individual pairs and balanced case and control groups.

Data on demographic characteristics, including sex, age, comorbidity, medical records, and clinical outcomes within follow-up period, were collected. The diagnosis of comorbidities was based on confirmed medical records from specialists in cardiovascular, chest, neurologic, gastrointestinal, hematology, endocrinology, nephrology, rheumatology, and psychology at Hualien Tzu Chi Hospital. Patients with lower socioeconomic status (SES) or physical or mental disabilities were identified and recorded as a disadvantaged subgroup.

### 2.3. Primary Outcome

The primary outcome was the occurrence of osteoporotic vertebral fractures (OVFs) over the thoracolumbar spine during the follow-up period. OVFs were defined as fractures caused by minor trauma, indicating bone fragility secondary to osteoporotic changes in the weight-bearing spine. According to the literature, OVFs were confirmed by a vertebral height loss of more than 25% compared with adjacent intact vertebra on lateral plain films, sagittal CT, or MRI.

### 2.4. Secondary Outcomes

The secondary outcomes included the clinical intensity of pain, which was measured using the Visual Analog Scale (VAS), and the effect of OVFs on QOL, which was measured using the Oswestry Disability Index (ODI). Specifically, VAS scores were documented before treatment (baseline) and at 1 week, 1 month, 3 months, 6 months, and 12 months after the index date. Consulting previous studies on the relief of symptoms related to spinal lesions, the present study used VAS score differences between baseline and post-treatment to quantify the effects of the ESI intervention and of conservative treatment alone [34,35,36,37,38,39]. A pain reduction of more than 50% compared with baseline VAS scores was defined as an ideal response, whereas a reduction of less than 50% was considered fair [39,40,41,42]. The ODI is a patient-completed questionnaire that is used to assess functional status and QOL in patients with acute or chronic low back pain. The ODI comprises 10 items that pertain to functional limitations in various activities of daily living, with scores ranging from 1 (best) to 100 (worst). The ODI consists of domains related to pain intensity, personal hygiene, walking, sleeping, social life, sex life (optional), and travel. Each domain has six possible responses, scored from 0 to 5, with only one ODI score required for each domain [43]. Given that the sample comprised Taiwanese people, the Chinese-adapted version of the ODI questionnaire was applied to assess native Chinese speakers through in-person or telephonic interviews, which typically took approximately 3–5 min to complete [44]. An ideal improvement in the disability index was defined by a 10-point decrease, corresponding to an average of a one-point decrease in each domain of QOL.

### 2.5. Adherent to Returning Follow-Up

During the follow-up period, each OPD visit was investigated separately to determine the purpose of the visit through interviews, questionnaires, or telephonic interviews. The reasons for OPD returns were categorized according to the chief complaint and major disorder at the time of the visit. The number of visits for lumbar spinal disorder and other comorbidities was recorded separately. Patients were also categorized on the basis of the frequency of OPD visits within the follow-up period. Those with more than two visits per 12 months for lumbar spinal disorder were considered actively adherent to follow-up for this condition. Given the average number of comorbidities in the database (4–5 diseases) and the typical prescription duration for comorbidities (2–3 months per prescription), patients with more than 12 visits per 12 months for all comorbidities other than the lumbar spinal disorder were considered actively adherent to follow-up for other comorbidities. Therefore, the criteria for active and inactive adherence were further defined as follows: active adherent: >2 visits/12 months for lumbar spinal disorder and >12 visits/12 months for other comorbidities and inactive adherent: ≤2 visits/12 months for lumbar spinal disorder and ≤12 visits/12 months for other comorbidities. These criteria were used to segment patients by the frequency of OPD returns during the follow-up period.

### 2.6. Statistical Analysis

The ESI and non-ESI control groups were compared. Normality for continuous variables was evaluated using the Kolmogorov–Smirnov test. Categorical variables (age, sex, comorbidities, disadvantaged status, and OVF events) were analyzed using the chi-squared test or Fisher’s exact test, and continuous variables (age, total follow-up duration, VAS, ODI score, OPD visits, and OPD visits per 12 months) were analyzed using the Student’s *t* test and the Mann–Whitney test. Inter-observer and intra-observer agreement was assessed using the intraclass correlation coefficient. Incidence density rates (per 1000 person-month [PM]) of osteoporosis, for subgroups stratified by sex, age, comorbidities, SES (disadvantaged status), and pain-relieving effect, were calculated. All-cause mortality was considered a competing event in the analysis. Cox regression was applied to calculate the crude hazard ratios (cHR) and adjusted hazard ratios (aHR) of osteoporotic spinal fractures. Subgroup analyses were conducted by sex, age, comorbidities, SES, pain-relieving effect, and OPD visit rate to identify predisposing factors for osteoporotic spine fractures. All statistical analyses were performed using SPSS software (version 21.0; SPSS, Chicago, IL, USA). *p*-values of < 0.05 indicated statistical significance.

## 3. Results

### 3.1. Demographic Characteristics of Participants

Among the retrospective cohort of more than 10,000 annual visits to the orthopedic, neurosurgery, and pain outpatient clinics at a medical center, 3820 patients met the inclusion and exclusion criteria between 2020 and 2022 and were included in the analysis. Among them, 80 patients who had received ESIs for LS were extracted to form the ESI group. Subsequently, 64 patients in the ESI group were successfully matched to four control samples each using 1:4 PSM based on age, sex, comorbidities, and SES. The matched cases constituted the non-ESI control group (*n* = 256; Table 1). An analysis of baseline demographic data revealed comparable sex ratios (male patients 40.63% vs. 42.58%, *p* = 0.777) and mean ages (64.59 ± 8.8, 64.50 ± 9.1, *p* = 0.943) between the ESI and control groups. Most comorbidities had comparable incidence rates between the groups. Disadvantaged status was similarly distributed in both groups (disadvantaged 18.8% vs. 21.5%, *p* = 0.631).

### 3.2. Clinical Status

The baseline pain intensity level was comparable between the ESI and control groups (7.28 ± 1.32 vs. 7.14 ± 1.40, *p* = 0.452). Moreover, the ODI-indicated QOL at baseline was also comparable between the two groups (32.63 ± 6.85 vs. 31.94 ± 6.92, *p* = 0.479). The primary and secondary outcomes were tracked at each follow-up time point (Table 2). Although the total follow-up duration was similar in both groups (30.97 ± 19.7 vs. 33.06 ± 19.6, *p* = 0.446), the ESI group had less frequent OPD follow-up visits for lumbar spinal disorders than the control group (1.83 ± 2.45 vs. 7.05 ± 8.85, *p* = 0.001). Moreover, the OPD return rate of lumbar spine disorders (visits/12 months) among the remaining patients who were followed up significantly differed between the groups (1.77 ± 4.19 vs. 3.94 ± 5.92, *p* = 0.006). When patients were divided based on a cutoff of two visits per 12 months, the percentage of inactive OPD adherents for lumbar spine disorders was significantly higher in the ESI group than in the control group (76.56% vs. 60.16%, *p* = 0.015). However, for overall comorbidities, the OPD return rate, except for visits related to the lumbar spine, was not significantly different between the groups (14.09 ± 29.1 vs. 19.00 ± 49.9, *p* = 0.450). When the patients were divided according to a cutoff of 12 visits per 12 months, similar percentages of inactive OPD adherents for comorbidities were observed between the groups (82.81% vs. 74.61%, *p* = 0.168).

During the follow-up period in this matched retrospective cohort, 11 cases (5 in the ESI group and 6 in the control group) of OVFs were observed, with a higher incidence rate in the ESI group (2.52 vs. 0.71 1000-patient-month, *p* = 0.047). When enrolled cases from 1 week to 12 months post-treatment were tracked, the VAS score, representing pain intensity, was significantly lower in the ESI group than in the control group at 1 week (3.73 ± 0.88 vs. 4.45 ± 1.55, *p* = 0.001), 1 month (3.45 ± 1.25 vs. 5.93 ± 1.19, *p* = 0.001), and 3 months (4.28 ± 1.22 vs. 5.09 ± 1.32, *p* = 0.002) post-treatment. More than 70% (70.30%) of the patients in the ESI group reported an ideal pain-relieving effect after the intervention, significantly more than the control group (70.30% vs. 48.80%, *p* = 0.002). Although a trend of decreasing ideal response rates was observed over time, the ESI group maintained a significantly higher percentage of ideal responses up to 3 months post-treatment.

In addition to pain intensity (VAS), the QOL as measured by the ODI also demonstrated significantly greater improvement in the ESI group 1 week after initial treatment (22.75 ± 8.22 vs. 28.3 ± 7.73, *p* = 0.001). This improvement persisted until the third month after initial treatment (21.22 ± 5.01 vs. 28.26 ± 6.46, *p* = 0.005). However, no significant differences in pain or disability status were observed at 6 or 12 months after initial treatment

### 3.3. Cox Regression Analysis

A Cox regression model (Table 3) was employed to analyze the incidence of OVFs in both groups. ESI was the only variable that was a risk factor for osteoporotic fractures (crude HR: 3.487, 95% confidence interval [CI]: 1.064–11.427, *p* = 0.039). Moreover, the analysis included most of the baseline categorical variables such as sex, age subgroups, comorbidities, disadvantaged status, and pain-relieving effect. However, after all the parameters were adjusted in the regression model, only ESI was determined to be an independent risk factor for OVFs, for all subgroups stratified by all other baseline variables (adjusted HR: 4.596, 95% CI: 1.011–20.89, *p* = 0.048).

### 3.4. Subgroup Analysis

A subgroup analysis was conducted to examine the associations of all variables with the risk of OVFs after receiving ESIs (Table 4). The results revealed significantly higher rates of OVFs in patients with diabetes mellitus (DM) (HR: 8.03, 95% CI: 1.341–48.136, *p* = 0.023) and those from disadvantaged socioeconomic backgrounds (HR: 11.818, 95% CI: 1.064–131.261, *p* = 0.044). Furthermore, patients without chronic heart failure (CHF) (HR: 4.209, 95% CI: 1.218–14.542, *p* = 0.023), chronic obstructive pulmonary disease (COPD) (HR: 3.645, 95% CI: 1.112–11.952, *p* = 0.033), liver cirrhosis (LC) (HR: 3.343, 95% CI: 1.020–10.960, *p* = 0.046), dementia (HR: 4.304, 95% CI: 1.245–14.873, *p* = 0.021), neoplasms (CA) (HR: 3.901, 95% CI: 1.128–13.487, *p* = 0.032), or hip osteoarthritis (OA; HR: 3.487, 95% CI: 1.064–11.427, *p* = 0.039) were at a higher risk of OVFs after receiving ESI compared with those who did not receive the intervention.

Having a disadvantaged status (lower income or disabled; HR: 11.818, 95% CI: 1.064–131.261, *p* = 0.044), experiencing an ideal pain-relieving effect (≥50% VAS reduction than baseline; HR: 6.086, 95% CI: 1.114–33.261, *p* = 0.037), and returning to the OPD less than 12 visits per 12 months for comorbidities other than lumbar disorders (HR: 4.074, 95% CI: 1.017–16.319, *p* = 0.047) were also found to be independent factors for OVF among patients who received ESIs for symptom relief.

## 4. Discussion

### 4.1. Effectiveness of ESIs

Given the comparable baseline characteristics, including pain intensity (VAS; 7.28 ± 1.32 vs. 7.14 ± 1.40, *p* = 0.452) and functional status (ODI; 32.63 ± 6.85 vs. 31.94 ± 6.92, *p* = 0.479), between the ESI and control groups, the observed differences in outcomes can be attributed to the effects of the intervention. The ESI group had a significant reduction in pain scale (VAS) score one week after the index date, demonstrating the symptom-relieving effects of the treatment. Moreover, improvements in pain (VAS) and disability (ODI) persisted for up to 3 months following the initial treatment. These findings align with evidence-based guidelines from the American Society of Interventional Pain Physicians (ASIPP) [45,46,47], which strongly recommend transforaminal ESIs for pain reduction at 3 months postintervention based on moderate-quality evidence. Although some studies have suggested the superiority of transforaminal ESI over caudal ESI [48,49,50,51,52], a systematic review and meta-analysis concluded that both approaches yield similarly effective outcomes in managing lumbosacral radicular pain [48]. Another meta-analysis from the Cochrane Library revealed moderate-quality evidence supporting the effectiveness of ESIs in reducing leg pain and disability within the short term (2 weeks to 3 months), regardless of the approach method (caudal, interlaminar, or transforaminal) [53]. Therefore, the 3-month duration of ESI in this study is consistent with findings in the literature and is considered a reasonable clinical option for patients with relatively severe symptoms of LS. However, the observed differences in pain (4.78 ± 1.2 vs. 5.29 ± 1.2, *p* = 0.066) and disability (27.70 ± 5.4 vs. 27.12 ± 6.8, *p* = 0.701) between the groups at 6 months post-treatment decreased, indicating the limited effectiveness of general non-ESI management, such as oral medication, physical therapy, or lifestyle modification. Although the significant symptom-relieving effects of ESI were limited to 3 months postinjection, ESI’s lasting benefits contributed to improved QOL in various domains, including pain intensity, personal hygiene, walking, sleeping, social life, and travel function.

### 4.2. Adherent to Follow-Up at OPD

Total follow-up duration was comparable in both groups, ensuring adequate observation for outcome events. PSM ensured that the baseline distributions of comorbidities were similar between both groups. Consequently, the cumulative number of OPD return visits and return rates (visits/12 months) for all comorbidities were comparable in both groups, indicating equivalent compliance and motivation for regular follow-up. However, the patients in the ESI group exhibited a reduced tendency to return to ortho, neurosurgical, or pain OPDs for lumbar spine disorder follow-up compared with the control group. This, along with the significantly lower OPD return rate for lumbar spine disorders, could be attributed to decreased demand and motivation for medical care due to symptom relief or a perceived lack of need for further examination following the significant improvement achieved through ESI intervention. The observed improvement in pain and disability after ESI treatment may also explain the significantly higher percentage of inactive adherents in the ESI group than in the control group (76.56% vs. 60.16%, *p* = 0.015), which is also consistent with the rate of ideal pain relief (70.30% vs. 48.80%, *p* = 0.002) at 1 week following initial treatments.

### 4.3. Primary Outcome: Osteoporotic Spinal Fractures

During the observation period, the incidence of osteoporotic spinal fractures was significantly higher in the ESI group (5 cases, 7.81%) than in the control group (6 cases, 2.34%). This suggests a potential acquired risk associated with ESIs. A prospective study by Jain et al. revealed a significant decline in BMD at the spine and femur neck 3 months following an ESI, with a cumulative incidence of moderately to markedly low BMD reaching 20.17% over 6 months [54]. Additionally, a review published by the International Osteoporosis Foundation and National Osteoporosis Foundation in 2021 highlighted the potential for ESIs to cause bone loss, particularly when volumetric BMD is measured [55]. Moreover, a retrospective analysis by Mandel et al. concluded that each successive LESI increased the risk of vertebral body fracture by a factor of 1.21 (95% CI, 1.08 to 1.30, *p* = 0.003) after adjustment for covariates [28]. Additional retrospective studies have examined the relationship between ESIs and fracture risk, providing partial support for the underlying mechanisms of osteoporotic spinal fractures [26]. For example, a retrospective study by Kim et al. in postmenopausal women with lower back pain revealed that patients receiving over 10 ESIs with a cumulative triamcinolone dose exceeding 200 mg exhibited lower BMD, suggesting a potential risk of osteoporotic spinal fractures in individuals with osteoporosis or osteopenia [56]. Similarly, Kang et al. reported a negative effect of ESIs involving doses over 200 mg of triamcinolone annually on BMD in postmenopausal women treated for lower back pain [57]. The narrative review of the literature by Rosati et al. highlighted the systemic effects of corticosteroids as the most common complications of ESI, including the potential risks of osteoporotic fractures and infection [58]. Although the focus of Okano et al.’s study was on patients’ post-lumbar fusion, their findings revealed that ESIs can have varying effects on vertebral BMD in the lumbosacral spine region, with the sacral alae being particularly sensitive to the intervention [59].

### 4.4. Pathophysiology of Osteoporotic Spinal Fractures Following ESIs

Several pathophysiologic mechanisms have been found to contribute to the fracture risk associated with ESIs. From an endocrinological perspective, alternations in bone remodeling activity, as evidenced by changes in bone remodeling markers, such as serum carboxy-terminal cross-linked telopeptides of type 1 collagen (sCTX), have been observed following a single ESI intervention [60]. Clare et al. reported an early and substantial reduction in bone formation markers following ESI, resulting from a significant reduction in serum cortisol levels, which subsequently led to decreases in the markers of bone formation such as osteocalcin (OC) and procollagen type-1 N-terminal propeptide (P1NP) by approximately 21% and 22%, respectively, within 1 week of the injection. The persistence of suppressed markers for over 12 weeks suggests a prolonged effect on bone formation processes [61]. Moreover, a study focusing on BMD changes post-ESI revealed a significant decline in hip BMD by 0.018 g/cm^2^ at 6 months, exceeding the decline observed in an age-matched control population. This decline was accompanied by an increase in bone-specific alkaline phosphatase, indicating heightened bone remodeling activity, although the increase in the C-telopeptide of collagen I (CTX) was not significant. Furthermore, an increase in bone remodeling activity was observed, as evidenced by an increase in bone-specific alkaline phosphatase and CTX (54). Overall, the significantly higher incidence rate of OVF observed in the ESI group warrants further investigation to confirm the risk among specific patient populations.

### 4.5. Cox Regression Model

In the Cox regression analysis, although several characteristics such as female sex, older age, diabetes, asthma, chronic kidney disease, depression, disadvantaged status, and inactive adherence were associated with higher incidence rates of OVCFs, only receiving ESI intervention was identified as an independent risk factor when all parameters were adjusted for. As expected, female sex and older age groups were identified as risk factors, consistent with previous findings [27]. However, due to the relatively small number of events, the findings had insufficient statistical significance despite the obvious differences in incidence rates between subgroups and the reference group. In this cohort model, only the most distinguishable variable with high specificity could be determined to be significantly correlated to the outcome event of an OVF.

### 4.6. ESIs Toward OVFs

The results of this study demonstrate a significantly higher risk of OVFs in patients receiving ESI for lumbar disorders, both before (crude HR: 3.487) and after (adjusted HR: 4.596) adjusting for baseline variables. These findings agree with those of several previous studies. For instance, a large population-based retrospective cohort study by Chen et al. indicated a 21% increase in fracture risk with each ESI, highlighting the potential for significant bone density reduction [27]. Additionally, a prospective study by Al-Shoha et al. in postmenopausal women revealed a significant decline in hip BMD and increased bone remodeling markers after a single ESI, suggesting that even a single injection can have a measurable impact on bone health [60]. Although this effect may be due to the systemic circulation of corticosteroids, as evidenced by the decline in BMD at distant sites, the local deposition of corticosteroids may also compromise bone density in the lumbar spine region, potentially leading to OVFs [60]. Therefore, the acquired risk of OVFs following ESI in the lumbar spine region is a concerning consideration, warranting cautious application of ESI procedures worldwide.

### 4.7. Effect of ESIs on Subgroups

Because ESI receipt was identified as an independent risk factor for higher osteoporotic spinal fracture by the Cox regression model, further subgroup analysis may be necessary to identify and quantify the acquired risk associated with each characteristic. The results of this study suggest that patients with DM may be particularly vulnerable to osteoporotic spinal fracture after receiving an ESI procedure.

ESIs can significantly affect BMD in patients, including those with diabetes, due to the systemic absorption of corticosteroids [55,62,63]. A recent review by Rosati et al. confirmed the systemic effects of corticosteroids, such as hyperglycemia and hypothalamic–pituitary–adrenal axis suppression, leading to a decrease in BMD and an increase in fracture risk [58]. Studies have demonstrated that corticosteroids can affect the bones through various pathways, including the stimulation of osteoclast-mediated bone resorption and reduction in osteoblast-mediated bone formation, leading to osteoporosis, particularly in the lumbar spine and femoral neck [64,65]. In patients with diabetes, the risk is compounded because corticosteroids can exacerbate hyperglycemia, further complicating bone health. Gómez-de-Tejada et al. concluded that three local cortivazol injections resulted in suppression of the corticotropic axis, which persisted beyond 21 days after epidural injection. Additionally, postprandial glucose levels were elevated for a longer duration in patients with diabetes than in those without, indicating a more prolonged effect on the diabetic population [65]. However, a study by Kim et al. focusing on older women with DM revealed no significant differences in BMD changes post-ESI between those with and without DM, suggesting that low doses of glucocorticoids might be used without the exacerbation of osteoporosis risk in this subgroup [65,66]. Given these findings, clinicians must consider risks to bone health when administering ESIs, particularly in patients with diabetes who are already at an increased systemic risk for osteoporosis and fractures.

### 4.8. Inconsistent of Follow-Up and Delayed Identification of Impaired Bone Quality

In the subgroup analysis conducted in this study, patients without several comorbidities, such as heart failure, COPD, gastric ulcer, LC, any neoplasm, or osteoarthritis of the hip (hip OA), exhibited a heightened risk of osteoporotic spinal fractures following ESI. This finding was consistent with the significantly elevated risk observed in patients who demonstrated nonadherence to outpatient follow-up for their comorbidities. Additionally, patients who reported ideal responses to ESI were also at a higher risk of fracture events. These results could be attributed to the tendency of patients without specific irreplaceable medical treatments for underlying diseases or patients with acute symptoms and disability to neglect regular outpatient follow-up. However, the relationship between the frequency of follow-up for medical services and the missed diagnosis of bone density loss in patients with lumbar degeneration is complex and influenced by various factors. Consistent follow-up is crucial for the early detection and management of BMD loss, which is often associated with lumbar degeneration and predisposes individuals to osteoporotic spinal fractures [67,68]. Studies have demonstrated that patients with lumbar degeneration who fail to adhere to regular follow-up are at a higher risk of undiagnosed BMD loss, leading to an increased incidence of fragility fractures and other complications [69,70]. For instance, a study on patients with lumbar degeneration revealed a significant correlation between elevated levels of bone turnover markers, such as PINP, BALP, OC, and β-CTX, and lower BMD as well as a heightened risk of fragility fractures, underscoring the importance of regular monitoring to prevent long-term complications [71]. Additionally, the presence of spinal degenerative disease can hinder the accurate measurement of spinal BMD, necessitating the use of alternative sites, such as the femoral neck, for monitoring because degenerative changes may lead to erroneous spinal measurements [72]. The complexity of these factors underscores the importance of frequent follow-up visits to ensure accurate assessment and timely intervention. Baseline BMD, anti-TNFα treatment, and disease activity markers were predictive of BMD loss and subsequent osteoporotic fractures in patients with spondyloarthropathies, highlighting the need for regular follow-up to monitor these parameters and adjust treatment accordingly [69]. In postmenopausal women, higher lumbar and femoral neck BMD were associated with lumbar disk herniation, indicating that BMD assessment during follow-ups can aid in the identification of patients at higher risk and guiding appropriate interventions [73]. Additionally, the Dubbo Osteoporosis Epidemiology Study demonstrated the significant effect of spinal degenerative disease on spinal BMD measurements, reinforcing the importance of regular follow-ups to accurately monitor BMD and prevent misdiagnosis [72]. Therefore, frequent and regular return follow-up visits for medical services may be indispensable in preventing the neglect of diagnosing bone density loss and the risk of osteoporotic fractures in patients with lumbar degeneration. These follow-ups enable the timely detection, accurate assessment, and appropriate management of BMD-related complications. Furthermore, the subgroup analysis revealed that disadvantaged status in the form of lower SES or disabled status was strongly associated with OVCF risk (HR: 11.818, 95% CI: 1.064–131.261, *p* = 0.044); disadvantaged status had the highest hazard ratio among all variables with respect to which the sample was segmented. Several factors could plausibly explain this finding.

### 4.9. Disadvantaged Status and Risk of OVF Following ESIs

Previous studies have consistently demonstrated a heightened risk of osteoporotic fractures, including spinal fractures, among individuals with lower SES. Factors such as limited access to health care, lower educational attainment, and reduced income contribute to this disparity. For instance, a Korean study revealed a significantly higher prevalence and incidence of osteoporosis and OVFs among individuals receiving Medical Aid compared with those covered by National Health Insurance [74]. Similarly, research in Australia demonstrated a clear inverse relationship between SES and fracture incidence, with individuals in the lowest SES quintile experiencing fracture rates 2–6 times higher than those in the highest SES quintile [75]. In Spain, low income, rural childhood residence, and low education levels were identified as significant risk factors for fractures, including hip fractures, which often coincide with spinal fractures due to similar underlying osteoporotic conditions [76]. Furthermore, disparities in access to diagnostic tools such as dual-energy X-Ray absorptiometry (DXA) scans, which are crucial for early detection and management of osteoporosis, are evident. Women with higher education and income levels are more likely to undergo DXA scans and receive timely interventions to prevent fractures [77]. In the United States, financial, personal, and structural barriers exacerbate these disparities, affecting access to orthopedic specialty care and timely treatment [78]. Although some studies, such as one conducted in Nottingham, have not found a direct relationship between SES and the proportion of fracture types or osteoporosis diagnoses among patients attending fracture clinics, individuals from deprived areas were less likely to attend bone density scan appointments, indicating a gap in follow-up care [79]. Collectively, these studies underscore the critical need to encourage patients from disadvantaged backgrounds to undergo regular OPD follow-up after symptom relief through ESI interventions. Preventive measures and early interventions can significantly reduce the incidence of fractures and their associated costs. Pharmacologic treatments, including bisphosphonates and emerging therapies such as stem cell treatments, exhibit promise in improving bone regeneration and reducing fracture risks [80,81]. Additionally, implementing health policies that address the socioeconomic determinants of osteoporotic spinal fractures is essential to ensure equitable access to preventive and therapeutic care across disadvantaged populations.

### 4.10. Future Work-Appropriate Protocol for ESIs for Thoracolumbar Degeneration in the Future

After gaining an insight into the vulnerability that comes with the effectiveness of functional improvement by each ESI as nonsurgical management for thoracolumbar degeneration, an individualized treatment approach for the management of thoracolumbar degeneration was made an urgent issue of concern. Future care protocols may focus on several aspects to prevent the rising OVF risks followed by cost and burden to the patient and the whole socioeconomic system. First, recognizing the inherent risk in patients who were vulnerable to subsequential OVF events is vital. Second, informing the potential OVF and encouraging regular follow-up for examination or prophylactic medication for patients receiving ESIs, despite the satisfactory improvement after ESI management. Third, for those who have received multiple ESIs in vain, detailed and individualized investigation should be made to identify the major contraindication against the patients from appropriate surgical treatment. Fourth, optimizing patient conditions to individualized surgical treatment, including endoscopic techniques, minimally invasive techniques, or radiofrequency ablation if patients cannot sustain implantation surgery. In summary, it is a crucial issue to build up an individualized treatment approach for the management of thoracolumbar degeneration to prevent cumulative risks of repeated failed ESIs.

### 4.11. Study Limitations

This study has several limitations. First, dual-energy X-Ray absorptiometry (DEXA) was not applied to investigate the BMD of patients in this retrospective study. Regarding the pre-investigations of DEXA, the current gold standard of diagnosis of osteopenia or osteoporosis may quantify the change in BMD and theoretically explain the connection between the ESI better, the universality of investigating BMD via DEXA remains low in real-world practice as the costs of the DEXA exam were not generally covered by the health insurance system in our current medical system. Therefore, if we only enrolled patients who had had BMD investigation in this retrospective study, there would inevitably be selection bias. Further, despite the strong correlation between the mitigated BMD and osteoporotic vertebral fractures (OVF) concluded in the previous literature, the majority of cost and burden come after the events of osteoporotic vertebral fractures (OVFs) rather than low BMD score at screening. Therefore, the incidence of recently occurred OVF events in the following duration could represent the risk and negative impact in both groups more realistically.

Second, the retrospective design would lack a comparable control arm. To address this, the study employed propensity score regression to quantify the potential influence of underlying variables that may confound the assessment of impaired bone quality. Meanwhile, some patients with undetected poor BMD may be enrolled due to the relatively low diagnostic rate of osteoporosis or osteopenia in real-world practice, the proportion of undetected impaired bone quality would be equally distributed in both the ESI group and the control group after being successfully matched by most demographic characteristics including sex, age, comorbidity, income status, and the severity of the diseases by Visual Analog Scale (VAS) and the Oswestry Disability Index (ODI). Therefore, the incidence rate of OVF during the follow-up duration of the ESI group and the comparative matched group would represent a realistic negative impact and risk even without the investigation of change in BMD.

Third, ESI is a technically demanding procedure, and the efficacy of symptom relief may vary among physicians. To address this, the study design only included patients who received injections from qualified pain physicians with at least 5 years of experience, ensuring a high level of technical proficiency. Additionally, the injected component must have contained more than 4 mg of corticosteroid to exclude the heterogeneity arising from patients who received ESI with lower doses of corticosteroids.

Fourth, the relatively low incidence of the event and small case numbers in certain subgroups hindered the ability to perform a multivariate regression model to adjust for confounding factors within each subgroup. However, the significant findings from the crude subgroup analysis consistently elucidated the relationship between the acquired risk of osteoporotic spinal fracture following ESI and specific patient characteristics. Furthermore, another population-based cohort study conducted by the authors confirmed that patients with lower socioeconomic levels or those who did not adhere to regular reassessment at outpatient clinics due to underlying comorbidities were at a higher risk of osteoporosis

## 5. Conclusions

Although ESIs have gained widespread popularity as a nonsurgical treatment option for moderate to severe LS, certain patient characteristics may increase the risk of subsequent osteoporotic fracture and impaired QOL. Consequently, careful consideration of these factors is crucial for informed decision making by clinical physicians.

## Figures and Tables

**Table 1:** Baseline demographic characteristic of patients.

	Epidural Steroid Injection (*n* = 64)		Control Group (*n* = 256)		*p*-Value
	*n* or Mean	% or SD	*n* or Mean	% or SD	
Gender					
Male	26	40.63%	109	42.58%	0.777
Female	38	59.37%	147	57.42%	
Age (year)	64.59	8.8	64.50	9.1	0.943
Age subgroups					
50–60	22	34.38%	96	37.50%	0.718
60–70	26	40.63%	90	35.16%	
>70	16	25.00%	70	27.34%	
Follow duration (Months)	30.97	19.71	33.06	19.61	0.446
Comorbidities					
DM	13	20.31%	64	25.00%	0.433
HTN	32	50.00%	115	44.92%	0.466
CHF	5	7.81%	20	7.81%	1.000
CAD	6	9.38%	31	12.11%	0.541
COPD	3	4.69%	11	4.30%	0.891
Asthma	13	20.31%	52	20.31%	0.465
GU	2	3.13%	26	10.16%	0.085
LC	4	6.25%	31	12.11%	0.262
CKD	4	6.25%	18	7.03%	1.000
Dementia	4	6.25%	44	17.19%	0.031 *
HOA	2	3.13%	10	3.91%	1.000
KOA	16	25.00%	76	29.69%	0.459
Depression	4	6.25%	12	4.69%	0.536
Disadvantaged status					
Disadvantaged (low-income or disabled)	12	18.80%	55	21.50%	0.631
Regular health insured	52	81.30%	201	78.50%	
Clinical status					
VAS	7.28	1.32	7.14	1.40	0.452
ODI	32.63	6.85	31.94	6.92	0.479

Note. DM, diabetes mellitus; HTN, hypertension; CHF, chronic heart failure; CAD, coronary artery disease; COPD, congestive obstructive pulmonary disease; GU, gastric ulcer; LC, liver cirrhosis; CKD, chronic kidney disease; HOA, hip osteoarthritis; KOA, knee osteoarthritis; VAS, visual analogue scale ODI, Oswestry disability index. * *p*-value < 0.05 as statistically significance.

**Table 2:** Clinical outcomes at following durations.

	Epidural Steroid Injection (*n* = 64)		Control Group (*n* = 256)		*p*-Value
	*n* or Mean	% or SD	*n* or Mean	% or SD	
Follow Duration (Months)	30.97	19.71	33.06	19.61	0.449
OPD returns for lumbar spine disorder					
Return (visits)	1.83	2.45	7.05	8.85	0.001 *
Return rate (visits/12 Months)	1.77	4.19	3.94	5.92	0.006 *
Inactive adherent (≤2 visits/12 Months)	49	76.56%	154	60.16%	0.015 *
Active adherent (>2 visits/12 Months)	15	23.44%	102	39.84%	
OPD returns for all comorbidities other than lumbar spine disorder					
Return (visits)	14.09	29.1	19.00	49.9	0.450
Return rate (visits/12 Months)	8.22	21.2	10.03	25.1	0.597
Inactive adherent (≤2 visits/12 Months)	53	82.81%	191	74.61%	0.168
Active adherent (>2 visits/12 Months)	11	17.19%	65	25.39%	
Outcome events	32	50.00%	115	44.92%	0.466
Osteoporotic vertebral compression fracture	5	7.81%	6	2.34%	0.032 *
Hip fracture	0	0.00%	0	0.00%	
Proximal humerus fracture	0	0.00%	0	0.00%	
Distal radius fracture	0	0.00%	0	0.00%	
Incidence rate (Event per 1000-patient-month)	2.52		0.71		2.52
Visual Analog Scale (VAS)					
Baseline	7.28	1.32	7.14	1.40	0.452
1 Weeks Post-Treatment	3.73	0.88	4.45	1.55	0.001 *
1 Months Post-Treatment	3.45	1.25	5.93	1.19	0.001 *
3 Months Post- Treatment	4.28	1.22	5.09	1.32	0.002 *
6 Months Post-Treatment	4.78	1.24	5.29	1.21	0.066
12 Months Post-Treatment	4.81	1.29	5.21	1.26	0.186
Pain reduction > 50% VAS (Ideal response)					
1 Weeks Post-treatment	45	70.30%	125	48.80%	0.002 *
1 Months Post-Treatment	42	65.63%	11	7.86%	0.001 *
3 Months Post-Treatment	12	37.50%	25	17.86%	0.048 *
6 Months Post-Treatment	7	30.43%	18	14.29%	0.037 *
12 Months Post-Treatment	6	28.57%	15	13.76%	0.109
Oswestry Disability Index (ODI)					
Baseline	32.63	6.85	31.94	6.92	0.479
1 Weeks Post-Treatment	22.75	8.21	28.30	7.73	0.001 *
1 Months Post-Treatment	19.05	3.29	28.52	5.99	0.005 *
3 Months Post-Treatment	21.22	5.01	28.26	6.46	0.005 *
6 Months Post-Treatment	27.70	5.43	27.12	6.79	0.701
12 Months Post-Treatment	25.24	6.79	25.93	6.87	0.674

* *p*-value < 0.05 as statistically significance.

**Table 3:** Risk of osteoporotic vertebral fracture.

	Event	PM	IR	Crude		Adjusted	
				HR (95% CI)	*p*-Value	HR (95% CI)	*p*-Value
Epidural steroid injection							
No	6	8464.31	0.71	Ref.			
Yes	5	1982.20	2.52	3.49 (1.06–11.43)	0.039	4.60 (1.01–20.89)	0.048 *
Gender							
Male	1	3934.13	0.25	Ref.			
Female	10	6512.39	1.54	5.94 (0.76–46.64)	0.090	3.79 (0.44–33.33)	0.227
Age							
50–60	2	3573.46	0.56	Ref.			
60–70	6	4024.81	1.49	2.68 (0.53–13.08)	0.227	1.60 (0.26–9.96)	0.615
>70	3	2848.24	1.05	2.00 (0.33–12.12)	0.453	1.72 (0.22–13.21)	0.604
Diabetes mellitus (DM)							
No	6	7905.11	0.76	Ref.			
Yes	5	2541.40	1.97	2.75 (0.84–9.02)	0.096	3.46 (0.68–17.57)	0.135
Hypertension (HTN)							
No	4	5865.31	0.68	Ref.			
Yes	7	4581.21	1.53	2.47 (0.72–8.44)	0.151	1.60 (0.34–7.47)	0.553
Chronic heart failure (CHF)							
No	10	9562.04	1.05	Ref.			
Yes	1	884.48	1.13	1.10 (0.15–9.03)	9.029	0.84 (0.07–10.60)	0.893
Coronary artery disease (CAD)							
No	10	9438.87	1.06	Ref.			
Yes	1	1007.64	0.99	1.03 (0.13–8.08)	0.978	0.44 (0.03–6.11)	0.541
Congestive obstructive pulmonary disease (COPD)							
No	11	10,078.45	1.09	Ref.		-	-
Yes	0	368.05	0.00	0.05 (0.00–121970.3)	0.685	-	-
Asthma							
No	10	10,148.70	0.99	Ref.			
Yes	1	297.80	3.36	4.19 (0.52–33.54)	0.176	4.11 (0.36–47.38)	0.257
Gastric ulcer (GU)							
No	8	8449.43	0.95	Ref.			
Yes	3	1997.22	1.50	1.86 (0.49–7.07)	0.366	1.88 (0.34–10.44)	0.472
Liver cirrhosis (LC)							
No	11	9723.78	1.13	Ref.	-	-	
Yes	0	722.73	0.00	0.04 (0.00–1285.99)	0.558	-	-
Chronic kidney disease (CKD)							
No	8	9323.12	0.86	Ref.			
Yes	3	1122.11	2.67	3.71 (0.98–13.99)	0.053	2.34 (0.40–13.80)	0.346
Dementia							
No	10	9735.09	1.03	Ref.			
Yes	1	711.42	1.41	1.40 (0.18–11.04)	0.747	0.71 (0.07–7.44)	0.777
Any malignancy/cancer							
No	10	8896.79	1.12	Ref.			
Yes	1	1549.71	0.65	0.60 (0.08–4.65)	0.621	0.33 (0.03–3.52)	0.356
Hip osteoarthritis (HOA)							
No	11	10,119.49	1.09	Ref.			
Yes	0	327.03	0.00	0.00 (-)	0.000		
Knee osteoarthritis (KOA)							
No	6	7682.05	0.78	Ref.			
Yes	5	2764.47	1.81	2.50 (0.76–8.23)	0.133	3.34 (0.66–16.91)	0.145
Depression							
No	10	9978.28	1.00	Ref.			
Yes	1	468.23	2.14	2.17 (0.28–16.96)	0.461	0.49 (0.04–5.92)	0.575
Disadvantaged status							
No	6	8412.06	0.71	Ref.			
Yes	5	2034.44	2.46	2.53 (0.74–8.66)	0.139	2.24 (0.48–10.45)	0.303
Pain relieving response							
Fair (VAS reduce ≤ 50%)	5	5177.03	0.97	Ref.			
Ideal (VAS reduce > 50%)	6	5269.48	1.14	1.17 (0.35–3.87)	0.798	2.44 (0.52–11.50)	0.258
OPD adherent for lumbar spine disorder							
Inactive (≤2 visits/12M)	9	7640.09	1.18	Ref.			
Active (>2 visits/12M)	2	2806.42	0.71	0.63 (0.13–2.94)	0.551	0.27 (0.04–1.96)	0.175
OPD adherent for other comorbidities							
Inactive (≤12 visits/12M)	8	8473.22	0.94	Ref.			
Active (>12 visits/12M)	3	1973.29	1.52	1.64 (0.43–6.31)	0.469	3.33 (0.58–19.00)	0.196

* *p*-value < 0.05 as statistically significance. Abbreviations: IR: incidence rate, per 1000-person months; PM: person-months. The disadvantaged status was categorized by the low-income level or handicapper approved officially by the Department of Social Welfare of local government. Model was adjusted by gender, age, socioeconomic status, pain relieving response and most of baseline comorbidities except for certain comorbidities with outlier due to no event outcome was observed during following duration, including, ankylosing spondylitis, congestive obstructive pulmonary disease, liver cirrhosis, and hip osteoarthritis.

**Table 4:** Risk of osteoporotic vertebral fracture after epidural steroid injection.

	Epidural Steroid Injection						HR (95% CI)	*p*-Value
	Yes			No				
	Event	PM	IR	Event	PM	IR		
Overall	5	1982.20	2.52	6	8464.31	0.71	3.49 (1.06–11.43)	0.039 *
Gender								
Male	1	703.79	1.42	0	3230.33	0.00	-	0.691
Female	4	1278.41	3.13	6	5233.98	1.15	2.76 (0.78–9.79)	0.116
Age								
50–60	1	598.03	1.67	1	2975.44	0.34	4.45 (0.28–71.16)	0.291
60–70	3	860.65	3.49	3	3164.16	0.95	3.80 (0.77–18.87)	0.227
>70	1	523.53	1.91	2	2324.71	0.86	2.32 (0.21–25.60)	0.453
Diabetes mellitus (DM)								
No	2	1592.06	1.26	4	6313.06	0.63	2.02 (0.37–11.01)	0.418
Yes	3	390.15	7.69	2	2151.26	0.93	8.03 (1.34–48.14)	0.023 *
Hypertension (HTN)								
No	2	1039.82	1.92	2	4825.49	0.41	4.56 (0.64–32.41)	0.129
Yes	3	942.38	3.18	4	3638.83	1.10	2.94 (0.66–13.12)	0.159
Chronic heart failure (CHF)								
No	5	1842.18	2.71	5	7719.84	0.65	4.21 (1.22–14.54)	0.023 *
Yes	0	140.02	0.00	1	744.46	1.34	-	0.795
Coronary artery disease (CAD)								
No	4	1875.19	2.13	6	7563.69	0.79	2.70 (0.76–9.57)	0.124
Yes	1	107.01	9.34	0	900.62	0.00	-	0.887
Congestive obstructive pulmonary disease (COPD)								
No	5	1896.53	2.64	6	8181.92	0.73	3.65 (1.11–11.95)	0.033 *
Yes	0	85.68	0.00	0	282.38	0.00	-	-
Asthma								
No	4	1875.19	2.10	6	8245.88	0.73	2.89 (0.81–10.23)	0.101
Yes	1	107.01	12.60	0	218.43	0.00	-	
Gastric ulcer (GU)								
No	4	1593.17	0.95	4	6856.24	0.58	4.34 (1.09–17.36)	0.038 *
Yes	1	389.03	1.50	2	1608.06	1.24	2.03 (0.18–22.36)	0.564
Liver cirrhosis (LC)								
No	5	1940.58	2.58	6	7783.20	0.77	3.34 (1.02–10.96)	0.046 *
Yes	0	41.62	0.00	0	681.11	0.00	-	-
Chronic kidney disease (CKD)								
No	4	1911.98	2.09	4	7412.42	0.54	3.93 (0.98–15.73)	0.053
Yes	1	70.22	14.24	2	1051.89	1.90	3.79 (0.34–41.81)	0.277
Dementia								
No	5	1852.64	2.70	5	7882.46	0.63	4.30 (1.25–14.87)	0.021 *
Yes	0	129.57	0.00	1	581.85	1.72	-	0.742
Any of malignancy/Cancer								
No	5	1846.95	2.71	5	7049.85	0.71	3.90 (1.13–13.49)	0.032 *
Yes	0	135.25	0.00	1	1414.45	0.71	-	0.853
Hip Osteoarthritis (HOA)								
No	5	1957.02	2.55	6	8162.48	0.74	3.49 (1.06–11.43)	0.039 *
Yes	0	25.18	0.00	0	301.84	0.00	-	
Knee osteoarthritis (KOA)								
No	3	1550.93	1.93	3	6131.11	0.49	3.95 (0.80–19.58)	0.092
Yes	2	431.28	4.64	3	2333.19	1.29	3.69 (0.62–22.18)	0.153
Depression								
No	4	1853.39	2.16	6	8124.88	0.74	2.93 (0.83–10.40)	0.096
Yes	1	128.81	7.76	0	339.42	0.00	-	0.594
Disadvantaged status								
No	2	1767.58	1.13	4	6676.11	0.60	2.02 (0.37–11.01)	0.418
Yes	3	214.62	13.98	2	1788.20	1.12	11.82 (1.06–131.26)	0.044 *
Pain relieving response								
Fair (VAS reduce ≤ 50%)	1	678.32	1.47	4	4479.72	0.89	1.61 (0.18–14.42)	0.670
Ideal (VAS reduce > 50%)	4	1284.89	3.11	2	3984.59	0.50	6.09 (1.11–33.26)	0.037 *
OPD adherent for lumbar spine disorder								
Inactive (≤2 visits/12M)	4	1731.39	2.31	5	5908.70	0.85	2.72 (0.73–10.13)	0.136
Active (>2 visits/12M)	1	250.82	3.99	1	2555.61	0.39	8.50 (0.53–137.19)	0.131
OPD adherent for other comorbidities								
Inactive (≤12 visits/12M)	4	1639.79	2.44	5	6833.43	0.73	4.07 (1.02–16.32)	0.047 *
Active (>12 visits/12M)	1	342.41	2.92	1	1630.88	0.61	1.67 (0.14–20.49)	0.690

Note. IR, incidence rate, per 1000-person months; PM, person-months; HR, Hazard ratio. * *p*-value < 0.05 as statistically significance. Abbreviations: The disadvantaged status was categorized by the low-income level or handicapper approved officially by the Department of Social Welfare of local government. The hazard ratios of several variables were omitted due to the insignificant finding of 95% confidence interval came from the subgroups without any event occurred.

## Data Availability

The data presented in this study are available on request from the corresponding author.
